# Superoxide Dismutase: A Predicting Factor for Boar Semen Characteristics for Short-Term Preservation

**DOI:** 10.1155/2014/105280

**Published:** 2014-03-05

**Authors:** Maja Zakošek Pipan, Janko Mrkun, Marjan Kosec, Alenka Nemec Svete, Petra Zrimšek

**Affiliations:** ^1^Clinic for Reproduction and Horses, Veterinary Faculty, University of Ljubljana, Gerbičeva 60, 1000 Ljubljana, Slovenia; ^2^Clinic for Small Animal Medicine and Surgery, Veterinary Faculty, University of Ljubljana, Gerbičeva 60, 1000 Ljubljana, Slovenia

## Abstract

Superoxide dismutase (SOD), total antioxidant capacity (TAC), and thiobarbituric acid reactive substances (TBARS) in seminal plasma were evaluated on the basis of receiver operating characteristics (ROC) analysis as predictors for distinguishing satisfactory from unsatisfactory boar semen samples after storage. SOD on day 0 correlated significantly with progressive motility (*r* = −0.686; *P* < 0.05) and viability (*r* = −0.513; *P* < 0.05) after storage; TBARS correlated only with motility (*r* = −0.480; *P* < 0.05). Semen samples that, after 3 days of storage, fulfilled all criteria for semen characteristics (viability > 85%, motility > 70%, progressive motility > 25%, and normal morphology > 50%) had significantly lower SOD levels on the day 0 than those with at least one criterion not fulfilled (*P* < 0.05) following storage. SOD levels of less than 1.05 U/mL predicted with 87.5% accuracy that fresh semen will suit the requirements for satisfactory semen characteristics after storage, while semen with SOD levels higher than 1.05 U/mL will not fulfill with 100% accuracy at least one semen characteristic after storage. These results support the proposal that SOD in fresh boar semen can be used as a predictor of semen quality after storage.

## 1. Introduction

Evaluating the fertility of sperm is economically important in breeding management. The modern boar industry worldwide is based on the use of artificial insemination (AI) of sows with mostly cooled semen. Semen is stored at 15–20°C for 1 to 5 days in the liquid state after dilution in an appropriate extender [1]. However, after short-term liquid storage, the quality of boar spermatozoa is diminished [2]. Additionally, differences between individuals in boar sperm longevity and survivability are well known [3].

The true index of fertility is the pregnancy and farrowing rate; however, both are expensive, time consuming, and influenced by factors extrinsic to the boar, such as sow quality and breeding management. While poor semen quality is a good indicator of reduced fertility, good semen quality (in terms of concentration, motility, and morphological normality) is not necessarily a warrant of acceptable fertility and long lasting semen viability [4]. Moreover, despite having normal or comparable sperm parameters in fresh semen, large differences have been noted in sperm parameters after short-term storage [5]. There is, therefore, a need for new sperm function parameter that would relate better to semen characteristics after storage and fertility.

Increasing evidences suggest that generation of reactive oxygen species (ROS) occurs during preservation of sperm [6]. Excessive ROS generation is detrimental to boar sperm cells and is associated with time-dependent decrease in motility, viability, and membrane permeability of spermatozoa during storage [7]. High sensitivity of boar semen to oxidative stress is due to the high content of unsaturated fatty acids in plasma membrane phospholipids and the relatively low antioxidant capacity of boar seminal plasma [8]. The mechanisms by which ROS disrupt the sperm functions are believed to involve the peroxidation of the polyunsaturated fatty acids present in the sperm plasma membrane [9].

Porcine seminal plasma is endowed with low molecular nonenzymatic and enzymatic defense mechanisms that can protect against ROS [10], with high quantities of superoxide dismutase (SOD) [11]. SOD protects spermatozoa by catalysing the dismutation of superoxide anions to hydrogen peroxide and oxygen, thereby protecting mature spermatozoa against excessive superoxide anion accumulation [9]. Owing to the high ability of boar semen for superoxide dismutation and lack of catalase-like activity in boar spermatozoa or seminal plasma [11] hydrogen peroxide is suggested to be the primary source of oxidative damage in boar sperm [12]. Interestingly, by cooling boar semen down to 5°C intracellular levels of ^·^O_2_ and H_2_O_2_ in sperm decrease [13]. As well, basal intracellular ROS formation is low in viable boar sperm of fresh and frozen-thawed semen [12].

The aim of our study was to determine whether oxidative stress biomarkers SOD, TAC, and TBARS in fresh semen could be helpful in predicting the quality of boar semen following short-term storage.

## 2. Materials and Methods

### 2.1. Semen Collection and Evaluation

Seventeen ejaculates were collected in spring from eight mature, healthy boars of various breeds (3 Slovenian landrace, 2 Slovenian large white, 2 Pietrain, and 1 Hibride line (54)) aging 12 to 24 months. All boars were fed commercial food (pellets) for AI boars and were housed in individual pens equipped with nipple drinkers to the European Commission Directive for Pig Welfare. Semen samples were collected routinely at the local AI centre (Ptuj, Slovenia) by the gloved-hand technique, using a clean semen collecting flask that filters out gel, dust, and bristles. Immediately after collection, semen was diluted 1 : 2 in Beltsville Thawing Solution (BTS, Truadeco, The Netherlands). While being analyzed, semen was kept at room temperature (20°C ± 1°C) for one hour in order to decrease chilling injury [14]. Prior to sperm motility analysis, 1 mL of diluted semen sample was incubated at 37°C for 8 minutes. A computer assisted sperm analyzer (Hamilton Thorne IVOS 10.2; Hamilton Thorne Research, MA, USA) with a Makler counting chamber (Sefi Medical Instruments, Israel) was used to evaluate motility and progressive motility. Sperm concentration was measured with a photometer (Photometer SDM 5, Minitüb, Germany) [15] while sperm viability was assessed using Hoechst staining [16]. The morphology of 200 spermatozoa was assessed in diluted semen samples after fixing in Giemsa stain [17].

Semen samples were then stored for 3 days in closed plastic containers in a thermal box. Temperature was controlled by calibrated thermometer and was kept at 16-17°C. Semen samples were constantly gently agitated in order to preserve the quality of stored semen [18]. The sperm characteristics of liquid-stored semen samples were evaluated after 3 days (72 h) of semen preservation, in the same way as that described above.

All samples were assessed in duplicates.

### 2.2. Preparation of Seminal Plasma and Analysis of Oxidative Stress Biomarkers

Extended semen was centrifuged at 818 ×g for 10 minutes at room temperature. The supernatant was removed and further centrifuged at 13,000 ×g for 15 min at 4°C to separate seminal plasma, which was then aliquoted and frozen at –80°C until assayed for TBARS, TAC, and SOD.

Semen samples were assayed for TAC by an automated biochemistry analyzer RX Daytona (Randox, Crumlin, UK), using a commercially available Total Antioxidant Status (TAS) kit (Randox, Crumlin, UK) that is based on the original method of Miller et al. [19]. The results are expressed as *μ*mol/L of Trolox equivalents.

SOD activity (U/mL) was determined spectrophotometrically with an automated biochemical analyser RX Daytona (Randox, Crumlin, UK), using the Ransod kit (Randox, Crumlin, UK) that is based on the original method of McCord and Fridovich [20].

Lipid peroxidation was measured by using the thiobarbituric acid (TBA) reaction for malondialdehyde (MDA) (TBARS assay Kit, Cayman Chemical Company). 100 *μ*L of seminal plasma from each sample was mixed with 2 mL of the TBA-TCA reagent (15%, w/v TCA; 0.375%, w/v TBA and 0.25 N HCl) and incubated in a boiling water bath for 60 min and subsequently cooled in an ice bath for 10 minutes. After cooling, the suspension was centrifuged at 1600 ×g and 4°C for 10 min. The supernatant was then separated, and absorbance was measured at 532 nm at room temperature over a period of 30 min after separation of supernatant. The assay was conducted in duplicate. The amount of TBARS produced (*μ*mol/L) was quantified against a standard curve created using MDA as standard (Tecan, Safir 2).

### 2.3. Statistical Analysis

#### 2.3.1. Correlation Analysis

Using Kolmogorov-Smirnov test, it was confirmed that data varies significantly from the pattern expected if the data was drawn from a population with a normal distribution; therefore, nonparametric tests were used in further evaluation.

Spearman rank correlation coefficient was used to determine the relationship between semen parameters on days 0 and 3. Statistical comparison of the results obtained on days 0 and 3 for each semen parameter was performed with the Mann-Whitney *U*-test. Statistical analyses were performed using Sigma Stat 3.5 (SYSTAT Software Inc., Illinois, USA). *P* < 0.05 was considered as significant.

#### 2.3.2. Diagnostic Evaluation

Diagnostic evaluation was performed to determine whether the boar semen quality after 3 days of storage could be predicted from oxidative stress markers values measured on the day of semen collection. Semen samples were divided into satisfactory (SAT) and unsatisfactory (UNSAT) groups according to individual semen parameter after 3 days of storage. Criteria for SAT semen samples were viability: >85%, motility: >70%, progressive motility: >25%, and normal morphology: >50%.

Samples were further categorized based on the number of satisfactory parameters that each individual semen sample achieved. Samples with all four parameters determined to be “satisfactory” were placed in Group 1; all the other samples were included in Group 2.

On day 0, samples were tested positive or negative according to SOD and TBARS. A positive test result (T+) was recorded when SOD or TBARS in seminal plasma was above the cut-off value. A negative test result (T−) was recorded when SOD or TBARS in seminal plasma was below the cut-off value. Together with the classification to SAT and UNSAT groups, four categories of results were obtained: true positive (TP), true negative (TN), false positive (FP), and false negative (FN). Diagnostic parameters, sensitivity, specificity, and positive and negative predictive values were calculated as described [21]. Sensitivity is indicated by the percentage of semen samples identified by the fresh oxidative stress semen parameter being unsatisfactory after 3 days of liquid storage. Specificity is a precentage of satisfactory samples after 3 days; samples were tested negative by fresh oxidative stress semen parameter. A positive predictive value (PPV) is the percentage of samples with a positive test result actually unsatisfactory after 3 days of storage. The negative predictive value (NPV) is the percentage of samples with a negative test result that are actually satisfactory after storage.

Receiver operating characteristic (ROC) curves were performed to determine the overall discriminating power of each semen variable. ROC curves plotted sensitivity versus 1-specificity for the complete range of cut-off points. All possible combinations of sensitivity and specificity that can be achieved by changing the breaking point are summarized by a single parameter, that is, the area under the ROC curve (AUC). A diagonal line in a ROC plot corresponds to a test that is positive or negative just by chance [22]. On the basis of the AUC for each semen parameter, we can determine whether the information is helpful in discriminating semen quality outcome after 3 days of storage or not. Helpful information about semen quality on day 3 is found when AUC close to 1 is observed together with statistical significance (*P* < 0.05).

ROC analysis was used to calculate the elective breaking point or cut-off value for oxidative stress markers in relation to semen quality after 3 days of liquid storage. Cut-off values for oxidative stress markers that can differentiate satisfactory from unsatisfactory semen samples after 3 days of storage were chosen to maximise the sum of sensitivity and specificity.

ROC analysis was performed using Analyse-it for Microsoft Excel (version 1.71) (Analyse-it Software, Ltd. http://www.analyse-it.com/, 2009).

## 3. Results

### 3.1. Sperm Characteristics on Days 0 and 3

Semen parameters were measured for fresh semen samples (day 0) and for semen samples after 3 days of liquid storage (day 3) (Table 1). All basic semen parameters differed significantly after 3 days of storage (*P* < 0.05). The concentration of spermatozoa did not change.

### 3.2. Correlation between Oxidative Stress Markers and Semen Parameters in Fresh and Stored Semen

Spearman correlation coefficients between oxidative stress markers (TAC, SOD, and TBARS) on day 0 and all semen parameters after 3 days of storage are shown in Table 2.

SOD in seminal plasma on day 0 showed a significant correlation with progressive motility (*r* = −0.686; *P* = 0.002) and viable spermatozoa (*r* = −0.513; *P* = 0.035) after 3 days of storage. A negative correlation near the level of significance was observed between SOD on day 0 and morphologically normal spermatozoa on day 3 (*r* = −0.423; *P* = 0.087). A negative correlation between TBARS on day 0 and motility on day 3 was also observed (*r* = −0.480; *P* = 0.054), whereas TAC on day 0 did not show any significant correlation with semen characteristics on day 3.

### 3.3. Correlation between Oxidative Stress Markers in Fresh and Stored Semen

Correlations between oxidative stress markers on days 0 and 3 were also seen. TAC in fresh semen correlated strongly with TAC and TBARS in stored semen (*r* = 0.918, *P* < 0.001 and *r* = −0.473, *P* = 0.054, resp.). TBARS on day 0 and day 3 showed negative correlation (*r* = −0.463) but it was not statistically significant (*P* = 0.059). No significant correlation was found between SOD on day 0 and oxidative stress markers in stored seminal plasma (*P* > 0.05).

### 3.4. Diagnostic Evaluation

Cut-off values (breaking points) and diagnostic parameters of oxidative stress markers on day 0 are presented in Table 3.

Regarding each individual parameter, SOD in fresh semen provided relevant information about progressive motility and viability after storage (AUC 0.86 and 0.85, resp.; *P* < 0.05). Optimal cut-off values of SOD for distinguishing stored semen according to progressive motility and viability were similar (1.22 and 1.26 U/mL, resp.).

SOD in fresh semen showed the highest predictive value for progressive motility after semen storage; 87.5% of semen samples had less than 25% progressive motility after 3 days of storage, whereas only 57.1% (PPV) of semen samples according to viability were actually unsatisfactory (less than 85% viable spermatozoa) after 3 days of storage. The NPV was high; 88.9% of semen samples with a concentration of SOD less than 1.22 had a satisfactory progressive motility above 25% after storage and 100% of semen samples with a concentration of SOD less than 1.26 U/mL had more than 85% viable spermatozoa after storage.

On the basis of lower AUC (AUC 0.70, *P* = 0.070), SOD provided less useful information in revealing semen quality outcome based on normal morphology.

**Figure 1:**
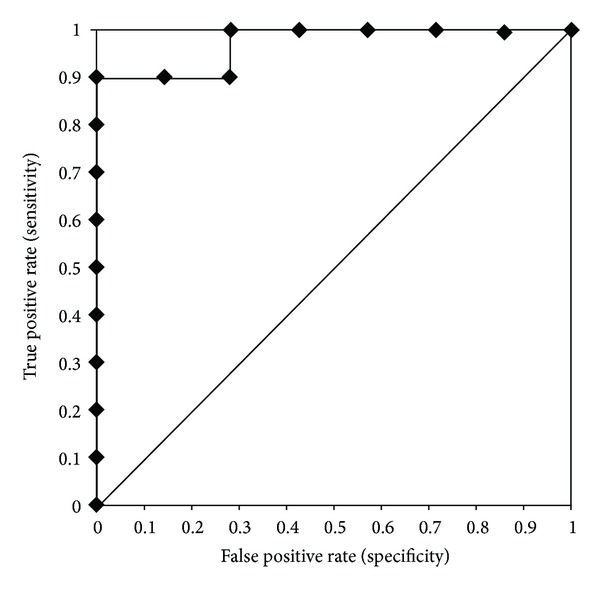
Receiver operating characteristics (ROC curve) for SOD on day 0 according to Group 1 and Group 2.

Semen samples classified in Group 1 fulfilled all criteria for satisfactory semen characteristics after storage, whereas semen samples in Group 2 had at least one unsatisfactory semen characteristic after storage. Based on ROC analysis, the threshold point of SOD on day 0 was 1.05 U/mL (Table 3). Sensitivity indicated that the concentration of SOD in fresh seminal plasma was less than 1.05 U/mL in 90% of the semen samples in Group 1, whereas 100% of the semen samples in Group 2 had ≥1.05 U/mL SOD in the seminal plasma. Predictive values were high; 100% of all semen samples with SOD ≥ 1.05 U/mL had at least one unsatisfactory semen characteristic (Group 2) after storage. Samples with concentrations of SOD in fresh seminal plasma of less than 1.05 U/mL, however, maintained 87,5% (NPV) accuracy of all criteria for satisfactory semen after storage (Table 3). The ROC curve for SOD on day 0 for distinguishing Group 1 from Group 2 is shown in Figure 1.

On the basis of AUC, TBARS provided less helpful information in discriminating semen quality outcome on the basis of motility (AUC 0.68; *P* = 0.089) (Table 3).

## 4. Discussion

Our study documented that SOD activity in fresh seminal plasma is a valuable indicator of porcine semen quality following liquid storage for 3 days. Current measures of semen quality are not always indicative of semen quality after storage [5] and do not predict accurately the ability of short-term stored spermatozoa to fertilize [21]. New markers of sperm function that would enable better prediction of fertilizing ability in boars have been sought [21, 23]. In this study, we have evaluated the possibility of using oxidative stress markers in fresh seminal plasma as predictors of the quality of boar semen submitted to short-term storage.

Interestingly, TBARS levels during storage in our study did not change significantly. In previous studies, contradictory reports are found concerning TBARS levels after liquid storage; Boonsorn et al. found even decreased levels of TBARS [24], whereas Kumaresan et al. reported increased TBARS levels [5]. These contradictory results from different studies in boars are difficult to explain. Even though TBARS assay is a well-establish method for screening and monitoring lipid peroxidation, the specificity of TBARS is questionable, because any sugar can yield a pink coloured product [25]. This could be the cause of contradictory results in different studies. Therefore, other methods for estimating lipid peroxidation like HPLC or estimation of isoprostanes, which are more specific, should be used [25].

However, there was a significant negative correlation in our study between levels of TBARS in fresh semen and semen motility following storage (*r* = −0.480; *P* < 0.05). Higher levels of TBARS in fresh semen could indicate that spermatozoa were under increased oxidative stress, which lead to a reduced motility after storage. This significant negative correlation between TBARS and semen motility could support other authors' suggestion that sperm motility is a sensitive indicator of oxidative stress and probably one of the first parameters affected [26]. However, despite the observed correlation noted above, based on ROC analysis, TBARS was not a valid marker for predicting boar semen quality after storage.

Total antioxidant capacity of seminal plasma was reduced during liquid storage which is in agreement with a study by Brzezinska-Slebodzinska et al. [8]. Am-In et al. reported that low TAC in seminal plasma is associated with lower storability of boar semen [27]. In the same study a significant negative correlation between TAC and percentage of normal sperm morphology was noted [27]. In our study levels of TAC in fresh semen samples did not correlate significantly with semen parameters after storage. These contradictory results could be due to experimental differences, including low numbers of ejaculates included in studies. Moreover, large numbers of sperm per dose could compensate for any fertility factor and mask any relationship with sperm quality [1]. Preselection of ejaculates by motility under commercial conditions tends to lead to lower variability in semen parameters, which could be manifested in stronger correlations than in the absence of preselection [28].

SOD activity in boar seminal plasma increased after three days of storage. Although SOD is a major antioxidant enzyme of boar seminal plasma [10], high levels of SOD activity are also found in boar spermatozoa [29]. Increased activity of SOD in seminal plasma could be related to the leakage of intracellular enzyme. Similar results are reported in bulls [30] and fowls [31], where SOD activity during storage decreases in spermatozoa but increases in seminal plasma. It was recently found that cooling boar semen from 15°C to 5°C may be primarily responsible for destabilization of sperm membranes (evaluated with 6-CFDA/PI), even though there was a decrease in intracellular levels of ^·^O_2_ and H_2_O_2_ [13]. Therefore, it could well be that increased SOD activity in seminal plasma in our study resulted from the leakage of intracellular enzyme from spermatozoa due to destabilization of sperm membranes as a result of liquid storage, without any concurrent detectable lipid peroxidation measured by TBARS. Further studies that would include measurement of membrane stability are needed to confirm the prediction mentioned above.

We have shown here that SOD in boar seminal plasma on day 0 correlates significantly with progressive motility (*r* = −0.686; *P* < 0.001) and viability (*r* = −0.513; *P* < 0.05) measured on the third day of storage. A negative correlation with levels of morphologically normal spermatozoa was near the level of significance (*r* = −0.423; *P* = 0.059). The role of SOD as a predictor of spermatozoa lifespan has already been suggested in humans, where a good linear correlation between the rate of spontaneous lipid peroxidation and SOD activity was observed [32]. Again in humans, a significant positive correlation was reported between recovery of motility after freeze-thawing and SOD content in sperm from the 90% gradient pellet containing highly purified mature sperm. However, in the same study there was also a significant negative correlation between motility after thawing and SOD content in the unfractionated sample [33]. In our study, SOD was measured in seminal plasma; therefore, an increase in SOD activity during storage could support the suggestion that increased SOD activity is a result of leakage of intracellular enzyme from sperm cells to seminal plasma.

The significant correlations between SOD in fresh seminal plasma and semen parameters after 3 days of storage led us to evaluate the ability of the former to predict semen quality on day 3. According to ROC analysis, it could be used to predict semen quality after 3 days of liquid storage in terms of progressive motility (AUC = 0.86; *P* < 0.001) and viability (AUC = 0.85; *P* < 0.001). We can predict with 88.9% certainty that fresh semen samples with SOD activity less than 1.22 U/mL will retain more than 25% progressive motility and, with 100% certainty, that fresh semen samples with activities of SOD less than 1.26 U/mL will retain more than 85% of viable spermatozoa after storage. TNF-*α* could also be used to predict the viability of boar semen following storage [21]. A cut-off value of 150 pg/mL would allow the prediction, with 92.35% certainty, that fresh semen samples with more than 150 pg/mL of TNF-*α* in seminal plasma will retain more than 85% of viable spermatozoa following 3 days of storage [21].

Diagnostic evaluation based on fulfilling four criteria for satisfactory semen characteristics after storage provided a higher prognostic value of SOD than similar evaluation based on individual semen parameter. It is important to know what percentage of samples with positive test result will not fulfil all criteria for satisfactory semen characteristics after 3 days of storage. The optimal cut-off value of SOD enabled the prediction, with 100% certainty, that fresh semen samples with SOD more than 1.05 U/mL will not fulfil criteria stated above. On the other hand, it can be predicted, with 87.5% certainty, that fresh semen samples with less than 1.05 U/mL will exhibit the required quality characteristics after storage. In our recent study, semen parameters for the prediction of boar semen quality following short storage were evaluated according to individual parameters. It was found that progressive motility could be predicted with at least 80% accuracy from progressive motility, normal morphology, and acrosome abnormalities of fresh semen samples [21].

## 5. Conclusions

SOD in seminal plasma of fresh boar semen was found to be a suitable predictive marker for progressive motility and viability following 3 days of storage. Moreover, SOD was a valuable indicator of semen quality following storage when looking at a combination of four standard parameters: motility, progressive motility, morphology, and viability. Therefore, SOD in fresh boar seminal plasma could be a reliable and simple test for predicting semen quality after 3 days of storage. Further studies are needed to evaluate SOD in fresh seminal plasma in relation to the pregnancy outcome in sows.

## Figures and Tables

**Table 1:** Summary of sperm parameters in AI dose (*n* = 17).

	Day 0		Day 3	
	Mean ± SD	Range (minimum–maximum)	Mean ± SD	Range (minimum–maximum)
Sperm concentration (million/mL)	279.0 ± 68.1	154.8–382.2	279.0 ± 68.1	154.8–382.2
Motility (%)	81.8 ± 8.2	67.0–94.3	68.9 ± 15.5	25.7–89.3
Progressive motility (%)	39.9 ± 5.2	30.0–46.7	26.3 ± 8.1	13.0–43.0
Morphologically normal form (%)	73.1 ± 9.6	53.2–84.7	55.8 ± 9.4	39.2–69.0
Viability (%)	93.2 ± 4.1	79.0–97.0	87.8 ± 4.4	76.0–93.5
Capacitated + acrosomal reacted (%)	13.1 ± 8.8	4.5–45.5	37.0 ± 11.4	22.5–66.5
TAC (*μ*mol/L)	808.8 ± 185.0	395.0–1140.0	770.9 ± 194.5	320.0–1100.0
TBARS (*μ*mol/L)	39.9 ± 5.4	30.8–48.7	39.0 ± 4.7	32.7–47.1
SOD (U/mL)	1.3 ± 0.7	0.5–2.9	2.7 ± 0.9	1.6–4.4

AI: artificial insemination; TAC: total antioxidant capacity; SOD: superoxide dismutase; TBARS: thiobarbituric acid reactive substances.

**Table 2:** Correlations (*r* value (*P*)) between oxidative stress markers in boar semen on day 0 and semen parameters after 3 days of storage.

Semen parameters and oxidative stress markers—day 3	Oxidative stress markers—day 0		
	TAC	TBARS	SOD
Motility	−0.135 (0.598)	−0.480* ** (0.049)**	−0.187 (0.466)
Progressive motility	−0.120 (0.639)	0.123 (0.632)	−0.686* ** (0.002)**
Morphologically normal form	−0.365 (0.145)	0.020 (0.936)	**−0.423^a^** ** (0.087)**
Viability	0.154 (0.546)	−0.309 (0.222)	−0.513* ** (0.035)**
Capacitated + acrosomal reacted	0.282 (0.266)	0.135 (0.598)	−0.346 (0.169)
TAC	0.918** ** (<0.001)**	**−0.473^a^** ** (0.054)**	0.223 (0.382)
TBARS	0.220 (0.387)	**−0.463^a^** ** (0.059)**	−0.186 (0.466)
SOD	−0.192 (0.454)	0.250 (0.326)	0.407 (0.107)

TAC: total antioxidant capacity; SOD: superoxide dismutase; TBARS: thiobarbituric acid reactive substances; *P*: statistical significance (*P < 0.05; **P < 0.001); ^a^near statistical significance.

**Table 3:** Cut-off values (breaking points) and diagnostic parameters of semen variables on day 0 and on day 3.

Variable—day 3	Breaking point	AUC (*P*)	Sens. (%)	Spec. (%)	PPV (%)	NPV (%)
	SOD (U/mL)—day 0					
Progressive motility	1.22	0.86 (0.0003)	87.5	88.9	87.5	88.9
Mor. normal form	1.26	0.70 (0.0697)	80.0	75.0	57.1	90.0
Viability	1.26	0.85 (0.0002)	100	76.9	57.1	100
Group 1/Group 2	1.05	0.97 (<0.0001)	90.0	100	100	87.5

	TBARS (*μ*mol/L)—day 0					
Motility	40.73	0.68 (0.0888)	60.0	66.7	42.9	80.0

AUC (ROC): area under the curve (receiver operating characteristics); *P*: statistical significance; Sens.: sensitivity; Spec.: specificity; PPV: positive predictive value; NPV: negative predictive value.
